# Discriminating Metabolic Health Status in a Cohort of Nursing Students: Protocol for a Cross-Sectional Study

**DOI:** 10.2196/21342

**Published:** 2020-08-28

**Authors:** Sarah L West, Holly Bates, Jessica Watson, Ingrid K M Brenner

**Affiliations:** 1 Department of Biology Trent University Peterborough, ON Canada; 2 Trent/Fleming School of Nursing Trent University Peterborough, ON Canada; 3 Department of Psychology Trent University Peterborough, ON Canada

**Keywords:** nursing, students, metabolic health, physical activity, sleep, nutrition, stress

## Abstract

**Background:**

Obesity is currently a worldwide health crisis. Nurses are integral members of the primary health care team and have an important role in managing obesity and administering physical activity (PA) for patients. However, research shows that nurses tend to be overweight or obese, have poor metabolic health, and do not meet PA recommendations. This is problematic because PA is linked to both physiological and psychological well-being and may also influence how nurses counsel their patients. Nursing students are the next generation of nurses; however, there is limited research examining PA (among other lifestyle factors) and metabolic health in nursing students.

**Objective:**

The goal of this research is to examine multiple lifestyle factors (including PA, nutrition, sleep, and stress) and determine whether these factors are associated with metabolic health in full-time undergraduate nursing students.

**Methods:**

An estimated 320 nursing students (18 years of age and older) will be assessed for their metabolic health. Metabolic status will be determined by measuring body mass index (BMI), waist-to-hip ratio (WHR), body fat percentage [skinfold measures (FitSystems Inc)], resting blood pressure [automated oscillatory (Omron Healthcare Inc)], and fasting blood glucose (glucometer). Lifestyle factors will also be measured, including PA and sleep [the International Physical Activity Questionnaire (IPAQ) and 7-day accelerometry (wGT3X-BT, Actigraph LLC)], nutrition [3-day diet log (Nutritionist Pro, Axxya Systems)], and stress [the Depression Anxiety Stress Scale, heart rate variability assessments, and salivary cortisol (ELISA, Eagle Biosciences)]. The association between metabolic status and PA, sleep quantity and quality, nutrition, and stress will be examined by linear regression analyses. Differences by year of study in metabolic health status, PA, sleep, nutrition, and stress will be examined by 1-way analyses of variance (ANOVAs). To determine the ability of PA, sleep, nutrition, and stress to discriminate prevalent overweight and obesity or poor metabolic status, logistic regression and receiver operating characteristic (ROC) curves will be constructed. Statistical analyses will be performed in Stata (version 16.1, StataCorp LLC).

**Results:**

Based on pilot data, we believe senior nursing students will have worse metabolic health (ie, higher BMI and WHR, increased body fat percentage, higher blood pressure, and increased fasting blood glucose) compared to first-year students. We hypothesize that poor PA participation, poor sleep quantity and quality, increased food intake, poor nutrition, and increased stress will be associated with worse metabolic health in full-time nursing students. The study received funding in February 2020. Due to the coronavirus disease 2019 (COVID-19) pandemic, work on this study has been delayed. We are currently completing our application for institutional research ethics approval. Data collection is projected to begin in January 2021, with data collection and analyses expected to be completed by May 2022.

**Conclusions:**

This study will be the first published research to examine the relationship between lifestyle choices and metabolic status in nursing students attending a Canadian institution. More importantly, the results of this study will support the development of an informed intervention that will target the identified lifestyle factors, improving the physiological and mental health and well-being of nursing students.

**International Registered Report Identifier (IRRID):**

PRR1-10.2196/21342

## Introduction

### Background and Rationale

Obesity is a current international epidemic [1], and it has become a global priority to reduce the burden of obesity [2]. Primary health care workers, which include registered nurses (RNs), interact closely with patients and therefore have an important role in managing an individual's obesity and associated secondary diseases. The critical role of nurses as agents of change in addressing the issue of obesity has been highlighted [3]. Unfortunately, research suggests that RNs themselves have a high level of obesity [4,5], more than the general population [6]. In addition to high rates of obesity, RNs do not meet international recommendations of 150 minutes per week [7] of moderate to vigorous physical activity (PA) [8,9]. One study reported that less than 30% of RNs sampled engaged in moderate-intensity PA [10]. Another study found that average body mass index (BMI) placed RNs (n=400) in the overweight category (>27 kg/m^2^), and many had elevated waist circumference [11]. Furthermore, only 23% met the recommended PA guidelines [11]. Overall, research demonstrates that RNs tend to be overweight or obese and are not meeting exercise recommendations.

High obesity rates and sedentary behavior in RNs are particularly concerning for two reasons. First, a nurse's own behavior may influence their nursing practice. Indeed, RNs who exercise regularly are more likely to promote PA to their patients compared to those who are sedentary [10]. In addition to potentially impacting patient care, obesity and sedentary behavior in RNs are concerning because nurses are not optimizing their own physical and mental health. RNs who are overweight or obese suffer from impaired cardiovascular and metabolic health [11]. As well, the nursing profession is associated with high levels of psychological stress [12,13] and high rates of depression compared to the general population [14,15]. It is widely recognized that PA is important for maintaining both physiological and mental health [16,17]. Physical inactivity may actually worsen mental health in RNs; in one study, physical inactivity was associated with psychological distress, even after adjusting for age, job demands, and job control, among other factors [18]. Sleep also contributes to mental health and overall well-being [19], and RNs report being fatigued and having poor sleep habits [20-22]. Shift work is associated with higher BMI and waist-to-hip ratio (WHR) [23], while participation in PA is associated with improved sleep in non-nurse populations [24-27]. Thus, obesity, poor PA, and reduced sleep are concerning in RNs, considering the negative effect of such lifestyle choices on nurses' physiological and mental health.

Nursing students are a unique population of primary care providers as they are still engaged in education, and therefore, there is an opportunity to educate students regarding the importance of lifestyle choices for themselves and their patients. Unfortunately, research to date indicates that similar to RNs, nursing students also have poor physical fitness behaviors [28], and they tend to participate in less PA compared to non-nursing students [29]. However, there is limited published data that objectively characterizes obesity and PA in nursing students. Therefore, we recently conducted a pilot study that examined PA and overweight and obesity status in a small cohort of nursing students attending a Canadian institution [30]. BMI and WHR were measured, and PA was objectively assessed via 7-day accelerometry in 43 full-time nursing students. Participants were classified into 3 groups based on their nursing program year: first year (n=13), second year (n=10), and third/fourth year (n=20). Mean body weight was higher in second and third/fourth-year students compared to first-year students. Similarly, BMI was higher in second-year students (23.7 ± 1.9 kg/m^2^) and third/fourth-year students (25.4 ± 2.5 kg/m^2^) compared to first-year students (20.3 ± 2.3 kg/m^2^; *P*=.005). Mean WHR was higher in third/fourth-year students (0.81 ± 0.06 cm) compared to first-year students (0.77 ± 0.04 cm; *P*=.04). Students were primarily sedentary (mean time spent sedentary was 81.7 ± 4.4%) and only engaged in an average of 9.9 ± 8.8 minutes of vigorous activity per day. Our pilot study results indicate that nursing students are highly inactive and that metabolic status based on BMI and WHR is worse in senior students [30].

We observed in our pilot study that nursing students are highly sedentary; however, it is important to note that PA is likely not the only factor that contributes to weight and obesity status in nursing students. For example, nutrition and food intake are directly correlated with BMI and WHR in RNs [31], and increased meal intake and poor nutrition are prevalent in shift workers [32]. As previously mentioned, poor sleep [33,34] and stress all likely contribute to worse metabolic health and overall well-being of nursing students. There is currently no study that examines the association of multiple lifestyle factors (including PA, nutrition, sleep, and stress) and metabolic health in a cohort of nursing students.

### Purpose and Objectives

The current study will expand upon previous research [30] and will characterize the factors that discriminate metabolic health of undergraduate nursing students attending a Canadian university. To this end, the objective of this study is to determine what factors are associated with metabolic health (measured by BMI, WHR, body fat percentage, blood pressure, and fasting glucose) in full-time undergraduate nursing students. More specifically, we will examine whether objectively measured PA, sleep, nutrition, or stress are associated with poor metabolic health status in a cohort of nursing students.

Based on pilot data [30], we believe that senior nursing students will have worse metabolic health (ie, higher BMI, higher WHR, increased body fat percentage, elevated blood pressure, and increased fasting blood glucose) compared to first-year students. We also hypothesize that poor PA participation, poor sleep quantity and quality, increased food intake, poor nutrition, and increased stress will be associated with worse metabolic health in full-time nursing students.

### Significance

The current study will be the first published research to examine the relationship between lifestyle choices and metabolic status in nursing students attending a Canadian institution. More importantly, the results of this study will be used to inform a lifestyle intervention for nursing students. To our knowledge, there are only 2 published PA and lifestyle intervention studies that target nursing students. In one study, nursing students and student midwives (N=182) from the UK were randomized into a control or an intervention group. Students in the intervention group received an education package and created a nutrition and physical activity plan. Following the 4-month intervention, the BMI of the overweight participants decreased, indicating that education and accountability may influence exercise participation in nursing and midwifery students [35]. In a second study, a convenience sample of 30 nursing students from a university in the United States who wanted to increase their PA levels were invited to participate in an exercise intervention. Students were asked to exercise for 30 minutes at least 3 times per week. Following one university semester of the intervention, students had reduced body fat percentages and BMIs, and higher levels of self-reported PA [36]. These studies support the idea that a lifestyle intervention can positively affect metabolic health status in nursing students.

The results of this study will directly inform a lifestyle intervention for nursing students that targets the improvement of their health and well-being, specifically addressing the most prominent factors associated with their poor metabolic health status. For example, if reduced PA, poor nutrition, and lack of sleep are all associated with worse metabolic health in nursing students, the developed intervention will focus on improving these outcomes. The development of an intervention will target the improvement of both the physical and mental health of nursing students. This intervention has the potential to influence future nursing practice since research highlights that RNs who are physically active will be more likely to counsel their patients to be physically active [10].

## Methods

### Overview of Study Design, Timeline, and Participant Selection

In this cross-sectional study, students enrolled in a school of nursing in an Ontario university in Canada will be invited to participate. A member of the research team will approach students in multiple ways, such as through classroom announcements, a post on the online learning system portal, posters, and word of mouth. Members of the research team who will approach and consent students will have no association with the students’ courses or academic work. Each year, the school of nursing enrolls approximately 200 students. We will aim to include approximately half of all students (ie, 80 students from each of the four years of nursing studies) to participate in the study (N=320). Our primary research objective is to examine multiple lifestyle factors (including PA, nutrition, sleep, and stress) and determine whether these factors are associated with metabolic health in full-time undergraduate nursing students; thus, we calculated our sample size based on our pilot data [30], which included an observed coefficient of determination value (R^2^) of 0.25 when we conducted a linear regression that examined whether participation in sedentary, light, moderate, and vigorous activity was associated with BMI. We determined that for a 2-tailed regression analysis with 4 predictors, a total sample size of 87 is required to provide us with statistical power of 0.95. Since we may have more than 4 predictors (participation in sedentary, light, moderate, and vigorous activity; nutrition, which may include total kilocalories (kcals); sleep, which may include total sleep; and stress), we also determined that for a 2-tailed regression analysis with 7 predictors, a total sample size of 103 is required to provide us with statistical power of 0.95. Thus, our sample size of 320 students is more than adequate. We chose to target a total of 320 students as we wanted to have a sufficient representation of those attending the nursing program.

**Figure 1:**
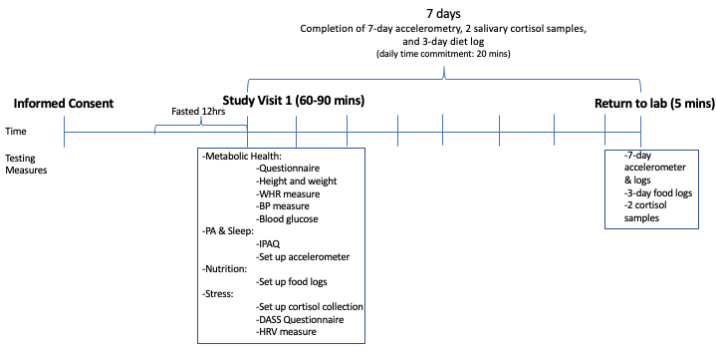
Illustration of the study timeline; BP: blood pressure, DASS: Depression Anxiety Stress Scale, HRV: heart rate variability, IPAQ: International Physical Activity Questionnaire, PA: physical activity, WHR: waist-to-hip ratio.

Ideally, we will recruit all participants during 1 academic year. If required, we will extend our recruitment and data collection over a second academic year. Specific inclusion criteria are (1) students enrolled in full-time nursing studies, (2) ≥18 years of age, and (3) signed informed consent. Only nursing students will be included in the current study (ie, there is no non-nursing student control group), since a comparison group is not required to address our research objective of examining discriminators of metabolic status in nursing students. Once the consent form is signed, students will meet with a member of the research team for their study visit (Figure 1). Only 1 study visit is required, which is estimated to take 60-90 minutes in total; therefore, this study involves minimal participant time commitment. Upon study completion, participants will receive a gift card to a popular snack or coffee location on the university campus.

### Assessments

#### Metabolic Health Measures

Metabolic health will be assessed by measuring obesity (BMI, WHR, body fat percentage), blood pressure, and fasting blood glucose, which are components of metabolic syndrome [37].

Participants will meet with a member of the research team and will complete a demographic questionnaire which includes information related to previous university or college studies, year of study in nursing, general health (ie, presence of any chronic diseases, use of medication), current living arrangements (ie, on-campus or off-campus with a commute), whether students are currently working or volunteering on the side, other health-related habits (such as drinking alcohol and smoking), as well as past history of PA participation. To further understand participation in PA over the past year, participants will complete the Modifiable Activity Questionnaire [38], which has been used to assess associations between physical activity and metabolic health in adults [39]. Participants will have their height (in centimeters) and weight (in kilograms) measured using a stationary research-grade stadiometer and scale. BMI will be calculated from the height and weight measurements: weight (kg)/height (m)^2^ [40]. Waist and hip measurements will be recorded as per the World Health Organization’s (WHO) guidelines using a Gulick tape measure [41]. At each site, 3 measurements will be taken, and they will be repeated if the measurements fall more than 1 centimeter away. The average value will be used to calculate the WHR: waist circumference (cm)/hip circumference (cm). Skinfold measures will be obtained using a Slim Guide skinfold caliper (FitSystems Inc). Measures will be obtained at the triceps, biceps, subscapular, suprailiac, and medial calf. Body fat percentage will be calculated using equations established by Durnin and Womersley (1974) [42].

Resting blood pressure (BP) will be measured using an automated oscillatory device (Omron Healthcare Inc) placed on the nondominant upper arm. Participants will be in a seated position for approximately 5 minutes prior to measurement. Two BP readings will be taken 60 seconds apart. An average of the 2 readings will be used.

Participants will be asked to fast (ie, to consume no food or drink other than water) for 12 hours prior to their study visit. Blood glucose will be measured using a standard glucometer; the third finger of the nondominant hand will be cleaned with an alcohol wipe and lanced using a single-use lancet. Blood will be collected on a test strip and measured via the glucometer.

#### Physical Activity and Sleep

Daily activity and participation in PA will be measured using 2 methods: (1) the International Physical Activity Questionnaire (IPAQ) and (2) objectively, via an accelerometer (wGT3X-BT, ActiGraph LLC).

Students will complete the IPAQ at their study visit. The IPAQ was developed in 1998 and is a validated questionnaire (across 12 countries) [43] that measures the PA and exercise that individuals complete as part of their everyday lives. It characterizes PA habits in the previous 7 days and considers physical activities of daily living, recreation, sport, and leisure time. The IPAQ contains 27 questions, is self-administered, and takes approximately 10 minutes to complete.

At their study visit, participants will be provided with an accelerometer (wGT3X-BT, ActiGraph LLC) to quantitatively measure activity and sleep. The wGT3X-BT accelerometer measures movement along 3 different axes: x-, y-, and z-axes. It is a valid PA measurement tool across many types and intensities of PA [44] that has been used previously to quantify PA in nurses [11] as well as in health professional students (including student nurses) [45]. Participants will wear the accelerometer on their nondominant hip (either clipped on their clothes or using a provided monitor belt) for 7 consecutive days [46], and on their nondominant wrist (using a wrist attachment) during sleep [47,48]. Participants will be instructed to wear the monitor during all waking and sleep hours, and to only remove the device during a shower or to partake in water-based activities. They will be asked to keep a detailed activity and sleep log, recording information such as when purposeful exercise occurs, sleep quality, and tiredness. Participants will be encouraged to maintain their regular activity levels and sleep habits.

After the 7-day data collection, the participant will return the accelerometer and the activity and sleep log. The accelerometer data will be downloaded using ActiLife software (version 6.13.3, ActiGraph LLC), with data further exported to Excel (version 16.4, Microsoft) to complete analyses. We will require a minimum of 4 days of accelerometer wear (confirmed by activity logs) for a participant's data to be considered valid [49,50]. We will not exclude prolonged bouts of inactivity that is accounted for because students may be attending class or might study for prolonged periods of time in a stationary position. If necessary, we will compare activity logs to the daily accelerometry activity records and exclude days where the accelerometer was not worn for >60 consecutive minutes for unaccounted reasons. The intensity of PA will be determined by counts per minute using the Freedson Adult algorithm [51]. These intensity cut-offs were used by our pilot study [30] and another study that examined daily PA via accelerometry in health professional students [45]. Data from the accelerometer will show the total kcals expended per day and the amount of time engaged in sedentary, light, or moderate/vigorous-intensity activity per day, as well as data on sleep times, sleep latency, and sleep efficiency [47].

#### Nutrition

During the same week as their accelerometry data collection, participants will complete a consecutive 3-day diet log (which includes 2 days of the week and 1 day of the weekend) and record all food intake. A 3-day diet log is commonly used to assess nutritional intake [52,53]. Logs will be analyzed with Nutritionist Pro software (Axxya Systems) for multiple outcomes, including total kcal intake and macro and micronutrient content.

#### Stress and Psychological Health

Levels of stress will be assessed using 3 methods: (1) a biochemical analysis of levels of salivary cortisol; (2) resting measures of heart rate variability (HRV), which can provide information on autonomic balance (sympathetic and parasympathetic tone); and (3) the Depression Anxiety Stress Scale (DASS).

For the biochemical analysis of salivary cortisol, participants will be asked to submit 2 saliva samples. Participants will collect 2 saliva samples (4-5 ml) in provided sample tubes, once during the morning and once during the evening on the first full day of their accelerometry collection week. Participants will record the day and time of their saliva sample collection, and will keep the salivary samples in the freezer until they are able to return their samples to the research laboratory. Samples will be transported back to the lab in a cooler. Cortisol, which is reflective of current stress levels, will be analyzed via ELISA (Eagle Biosciences).

For analysis of HRV, participants will have their heart rate monitored in lab at their study visit (following the collection of metabolic health measures) for 10 minutes in the supine position and 10 minutes in the standing position using a GPS sports watch (Polar V800). This information will be downloaded using Flowsync software (Polar) and analyzed using Kubios HRV software (Kubios Oy), which performs a fast Fourier transformation on the data collected to determine autonomic balance.

Students will complete the DASS at their study visit. The DASS is a widely used tool that has been validated in many populations to assess the emotional states of depression, anxiety, and stress [54]. There are reference values to which DASS scores can be compared [55]. It is important to note that the DASS does not measure clinical outcomes but rather the emotional states of anxiety, stress, and depression. The DASS is not intended to be used as a diagnostic tool for clinical cases. It considers how an individual has felt over the past week and is a 42-question scale that will take approximately 15 minutes to complete.

### Statistical Analysis

The demographic characteristics of the participants will be assessed using descriptive statistics. The association between metabolic status (BMI, WHR, body fat percentage, blood pressure, and fasting blood glucose) and PA outcomes, sleep quantity and quality, nutrition, and stress will be examined by linear regression analyses. To examine differences by year of study, 1-way analyses of variance (ANOVAs) will be conducted comparing metabolic health status, PA, sleep, nutrition, and stress. Bonferroni post-hoc testing will be conducted if significant group differences are present. To determine the association between PA, sleep, nutrition, and stress with metabolic health (ie, BMI, WHR, blood pressure, blood glucose), we will conduct linear regression analyses in all nursing students, and by year of study.

To determine the ability of PA, sleep, nutrition, and stress to discriminate prevalent overweight and obesity, participants will be placed into one of 2 groups: overweight or obese versus not overweight or obese, based on the WHO’s BMI and WHR cut-offs [40]. Logistic regression and receiver operating characteristic (ROC) curves will be constructed for each lifestyle variable [expressed as area under the ROC curves (AUROC) with 95% confidence intervals (CI)]. Similar analyses will be conducted using a composite indicator of metabolic status (incorporating obesity status, blood pressure, and fasting blood glucose). Additional analyses adjusted for age, gender, year of nursing study (including exposure to practicum), semester of school, and other potential confounding variables (such as prior physical activity habits) will be conducted as deemed necessary. Statistical analyses will be performed in Stata (version 16.1, StataCorp LLC).

## Results

Following the completion of this study, we expect to characterize the metabolic status, PA habits, sleep quantity and quality, food intake and nutritional status, and stress levels in nursing students attending a Canadian institution. We also expect to determine if PA, sleep, nutrition, or stress are associated with or discriminate obesity and metabolic status in nursing students.

The study received funding in February 2020. Due to the coronavirus disease 2019 (COVID-19) pandemic, work on this study has been delayed. We are currently completing our application for institutional research ethics approval. Data collection is projected to begin in January 2021, with data collection and analyses expected to be completed by May 2022.

## Discussion

This study will be the first to examine multiple factors associated with metabolic health in a cohort of nursing students attending a Canadian institution. This paper describes a cross-sectional study designed to characterize PA, sleep, nutrition, and stress of nursing students, and it will determine which of these factors are associated with metabolic health (BMI, WHR, body fat percentage, blood pressure, and blood glucose). Ultimately, the goal of our research program is to develop a supported and informed intervention that targets the improvement of the most prominent factors associated with poor metabolic health status in nursing students. This cross-sectional study is necessary as it will allow us to identify the factors impacting the metabolic health of Canadian nursing students and, therefore, to determine which factors should be targetted with an appropriate intervention. The use of “exercise as medicine,” among other lifestyle intervention approaches, has gained attention from researchers and health care professionals, since exercise is a potent method of reducing the prevalence of obesity and improving multiple health outcomes [56].

It is important to note that although we are measuring some important lifestyle factors (such as diet, physical activity, sleep, and stress), the current study does not consider all individual-environment interactions or structural environmental factors that may impact the metabolic health of nursing students. For example, one study in the UK that examined food intake in (non-nursing) university students suggested that university policies aimed at improving student diets should incorporate student engagement in food preparation and increased access to healthy, low-cost food [57]. Thus, socioeconomic status may play a role in the lifestyle and health of university students. Similarly, the distance a student lives in relation to their university campus may impact their health. One study of over 700 university students examined the presence of metabolic syndrome in students who were active commuters (ie, walked to campus) versus students who took motorized transport to campus (such as a car or bus); the prevalence of metabolic syndrome was almost 9% higher in the students who did not actively commute to school [58]. Therefore, students who are able to live close to campus (due to a variety of reasons, including socioeconomic ones) and engage in active commuting may have better metabolic health. Another factor that may influence participation in physical activity at the university level is the presence of a disability; one research study demonstrated that a large number of university students with an identified disability did not meet the WHO’s physical activity guidelines; therefore, special consideration of students who identify with a disability (and not just physical disabilities) is needed [59]. As well, higher academic achievement has been associated with better PA habits in university medical school students [60]. Thus, it is important to recognize that there are additional factors that are not being explored in the current study that may independently influence the metabolic health and lifestyle choices of university students, including nursing students. This study is a preliminary study characterizing some of the metabolic health and lifestyle factors in nursing students.

There are additional limitations to this study. This study will allow us to determine which factors correlate with the metabolic health of nursing students; however, we will be unable to determine whether these factors are directly causative (although it is likely that there are multiple factors that contribute to poor metabolic health of nursing students). Furthermore, we are not following students longitudinally; rather, we are examining the profile of the student population at one given time. Since we intend to use the results of this study to inform a lifestyle intervention, we believe these limitations are not problematic, as we are interested in learning which factors might be best to target with our intervention.

In conclusion, this study will provide valuable data describing the nursing student cohort at a Canadian institution. It will ultimately support an informed lifestyle intervention that will target the improvement of the physiological and mental health and well-being of nursing students, as well as education related to the importance of lifestyle choices for themselves and their future patients.

## Appendix

Multimedia Appendix 1 Peer review reports from Trent/Fleming School of Nursing Research Grant Program.

## Abbreviations

ANOVA: analysis of variance
AUROC: area under the receiver operating characteristic curve
BMI: body mass index
BP: blood pressure
COVID-19: coronavirus disease 2019
DASS: Depression Anxiety Stress Scale
HRV: heart rate variability
IPAQ: International Physical Activity Questionnaire
kcal: kilocalorie
PA: physical activity
RN: registered nurse
ROC: receiver operating characteristic
WHR: waist-to-hip ratio
WHO: World Health Organization
