# Supplementary material for: Discriminating Metabolic Health Status in a Cohort of Nursing Students: Protocol for a Cross-Sectional Study
Source: JMIR Res Protoc. 2020 Aug 28;9(8):e21342. doi: 10.2196/21342 (PMC7486670; doi:10.2196/21342)
Supplement: Multimedia Appendix 1 [file resprot_v9i8e21342_app1.docx]

**Reviewer A:
General comments
Overall, the paper is well written with a rigorous study design that is in alignment with the stated objective. Room to provide a more fulsome delineation of factors influencing metabolic health by addressing influencing factors beyond individual life style choices.** 

**Major comments
1. A more fulsome delineation of the issue presented is needed. The complexity and multifaceted of lifestyle choices should be addressed; specifically, some discussion of individual-environment interactions and structural environmental factors known or hypothesized to also have an impact is needed. Nursing and other health professional students content with health care and educational systems and structures that influence their health status. This can be addressed in the background, discussion, or limitation section of the study.**

RESPONSE: We thank Reviewer A for their helpful comment. We addressed this in the discussion, related to limitations of the study. We added the following paragraph, with additional new references (page 15-16):

“It is important to note, that while we are measuring some important lifestyle factors (such as diet, physical activity, sleep, and stress), the current study does not consider all individual-environment interactions and/or structural environmental factors that may impact the metabolic health of nursing students. For example, one study examined food-intake in (non-nursing) university students in the U.K., and suggested that university policy to improve student’s diets should incorporate student engagement in food preparation, as well as increased access to healthy low-cost food [Sprake, 2018 #11799]. Thus, socioeconomic status may play a role in lifestyle and/or health of university students. Similarly, the distance a student lives in relation to their university campus may impact their health. One study of over 700 university students examined the presence of metabolic syndrome in students who were active commuters (i.e., walking to campus) vs. students who took motorized transport to campus (car, bus etc.); the prevalence of metabolic syndrome was almost 9% higher in the students who did not actively commute to school [Garcia-Hermoso, 2018 #11805]. Therefore, students who have the ability to live close to campus (due to a variety of reasons, including socioeconomic) and engage in active commuting may have better metabolic health. Another factor that may influence participation in physical activity at the university level is the presence of a disability; one research study demonstrated that a large number of university students with an identified disability did not meet the World Health Organization’s physical activity guidelines, thus special consideration of students who identify with a disability (i.e., not just physical disability) is needed [Ubeda-Colomer, 2019 #11800]. As well, higher academic achievement has been found to be associated with better physical activity habits in university medical school students [Al-Drees, 2016 #11810]. Thus, it is important to recognize that there are additional factors that are not being explored in the current study that may independently influence metabolic health and/or lifestyle choices of university students, including nursing students. The current study is a preliminary-study characterizing some of the metabolic health and lifestyle factors in nursing students.”

**Reviewer AA:
General comments
Thank you for the opportunity to review this research protocol to examine the relationship between lifestyle choices and metabolic status in Canadian nursing students. As you have identified, there are a number of studies examining the relationship among practicing registered nurses, particularly in relation to shift-work, but there is less published about students of nursing. Encouraging healthy work and life habits during this period is very much part of preparing students for a successful nursing career. 

Specific comments
The methods are generally well described except for a number of potentially confounding factors that are not discussed in the protocol but which may be important to consider in order to achieve meaningful statistical results.**

RESPONSE: Thank you, please see specific responses below.

**Major Comments
1. Potentially confounding variables that are not fully described include:
a. The metabolic profile and physical activity habits of students prior to entering nursing may be relevant. Will this be part of demographic questioning?**

RESPONSE: Thank you for this question, yes, we will incorporate prior physical activity habits into the demographic questionnaire. We have also added the completion of the Modifiable Activity Questionnaire at their study visit, which is a reliable and valid questionnaire to recall past-year (longer term) physical activity habits. The manuscript now reads (page 9-10):

“Participants will meet with a member of the research team and will complete a demographic questionnaire which includes information related to previous university or college studies, year of study in nursing, general health (i.e., presence of any chronic diseases, use of medication), current living arrangements (i.e., on campus, or off campus with a commute), whether students are currently working and/or volunteering on the side, other health related habits (i.e., such as drinking alcohol and smoking), as well as past history of physical activity participation. To further understand participation in physical activity over the past year, participants will complete the Modifiable Activity Questionnaire [Vuillemin, 2000 #11798], which has been used to assess associations between physical activity and metabolic health in adults [Boucher, 2015 #11797].”

**b. It is not clear whether there are differences across first, second year etc. in the amount/type of practicum (time spent in School vs. time spent working in wards) Canadian nursing students are required to complete? Are students required to undertake shift-work during their practical experience placements for example?**

RESPONSE: Yes, there are differences in the amount of practicum that the students are required to complete per year of study. At the institution we are recruiting students from, each year of study involves increasing amounts of practicum time. First year students complete 72 hours, second year students complete 256 hours, third year students complete 320 hours, and fourth year students complete 600 hours. In fourth year, students may engage in shift-work where they mirror their preceptor. This is why we are interested in examining lifestyle factors and metabolic health by year of nursing study; since the number of practicum hours increase and may involve shift work in their senior year. In the demographic questionnaire, we will record which placements students are currently completing (how many hours per week), so that we can determine the type of placement experience the students were engaged in while completing data collection. Combined with our program knowledge, we can determine how much practicum vs. in-class time each participant is exposed to. We have taken this into consideration in the statistics section of our manuscript (page 14): “Additional analyses adjusted for age, gender, year of nursing study (including exposure to practicum), semester of school, and other potential confounding variables such as prior physical activity habits, will be conducted as deemed necessary. Statistical analyses will be performed in STATA (16.1, Texas).”

**2. In relation to the above, if high variation is present between years in potential confounders AND there are multiple comparisons being made across a range of measures, the sample size of approx. 80 students per year may not be adequate. How was the sample size calculated?**

RESPONSE: We designed the current study based on the results of our pilot study that examined BMI, WHR, and accelerometry in 40 nursing students (citation 30 in the manuscript). Note: the pilot study is published in abstract format in *Applied Physiology Nutrition and Metabolism*, and is currently under review in the full manuscript format in the sister journal *JMIR Formative Research*.

It is important to highlight the stated objective of the study: “The goal of this research is to examine multiple lifestyle factors (including PA, nutrition, sleep, and stress) and determine whether these factors are associated with metabolic health in full-time undergraduate nursing students.”

Therefore, using data from our pilot study, we calculated our sample size based on examining the association of lifestyle factors and metabolic health. On the basis of our pilot data, which included an observed R^2^ =0.25 when we conducted a linear regression that examined whether participation in sedentary, light, moderate, and vigorous activity was associated with BMI, we determined that for a two tailed regression analysis with four predictors a total sample size of 87 is required to provide us with statistical power of 0.95. Since we may have more than 4 predictors (participation in sedentary, light, moderate, and vigorous activity, nutrition which may include total kcals, sleep which may include total sleep, and stress), we also determined that for a two tailed regression analysis with seven predictors total sample size of 103 is required to provide us with statistical power of 0.95. Thus, our sample size of 320 students is more than adequate. We chose such a large sample size as we wanted to have sufficient representation of the students attending our Nursing school. We have added this information into the manuscript (page 8-9).

It is also important to note that we are interested in characterizing the metabolic health of nursing students (i.e., what is the physical activity, diet, stress, etc. of nursing students. What is the metabolic status of nursing students?). We understand that our study will not provide causative data (i.e., what factors cause worse metabolic health); as this is a cross-sectional study. Because we will also be examining differences by year of nursing study, we conducted a sample size calculation for this as well.

Using data from our pilot study, we calculated two effect sizes (one for BMI and one for light activity measured by accelerometry) using the observed means and standard deviations by year of study. On the basis of mean and standard deviation of BMI by year of study, the calculated effect size was 0.968. With four groups (first, second, third, and fourth year students), a total of 24 participants is needed to achieve statistical power of 0.96. On the basis of mean and standard deviation of light activity by year of study, the calculated effect size was 0.266. With four groups (first, second, third, and fourth year students), a total of 248 participants is needed to achieve statistical power of 0.95. Therefore, our large sample size of 320 (80 per year), satisfies our requirements; and provides us with some room for student drop-out or incomplete tests.

**3. The protocol requires a significant time commitment from students. In addition to time spent at the study site for testing and survey completion, students will also be responsible for the self-management of recording activity and sleep, food intake and taking saliva samples. The study aims to recruit approx. half of all students from each of the four years of the nursing studies program (80 per year of study). It is highly likely that some participants will not complete all parts of the study. What plans do the researchers have for managing this in regard to overall sample size and statistical analysis?**

RESPONSE: Thank you for your comment. We actually believe that the study involves relatively minimal time commitment from the participants (page 9: “Only one study visit is required, therefore this study involves minimal participant time-commitment.”). We have added the following sentence to the Methods (page 9) to clarify the time commitment of the lab visit: “Only one study visit is required, which is estimated to take 60-90 minutes total…”

After the in lab study visit, participants are only required to take 2 saliva samples, complete 3 days of food logs, and wear the accelerometer for 7 days (this involves minimal work, since the accelerometer is a device that is attached to clothing and the students go about their normal day), and complete a brief accelerometer log. It is estimated that 20 minutes per day over the 7 day collection period is needed. We have revised Figure 1 in the document to include the time commitment of the study.

To further encourage study completion, we have added an incentive. Upon completion of the study, the participant will be provided with a gift card for a popular snack and coffee location on the University campus. We have added the following sentence to the paper (page 9):

“Upon study completion, participants will receive a gift card to a popular snack/coffee location on the University campus.”

We conducted a pilot study (citation 30 in the document, discussed in the Background) where we measured physical activity via accelerometer for 7 days in nursing students. All students who took home the accelerometer completed the 7 day assessment, and we received no feedback from the pilot study that students found it cumbersome. Based on our pilot study, we are confident that students will complete all of the testing. If we find, in contrast to our pilot study, that students are not completing the 7 day accelerometery, as discussed above our large target sample size and provides us with some room for student drop-out or incomplete tests.

**Minor Comments
4. There are a large number of tests to administer to students attending the study site. Assuming the sample size is reached and/or exceeded, does the research team have adequate resources to deliver the instruments and undertake direct testing with students returning to the lab after seven days (two visits each student) and across the four years of the cohort (640 visits) within the time-frame of the funded project?**

RESPONSE: Thank you for your comment. As indicated by the study title “Discriminating Metabolic Health Status in a Cohort of Nursing Students: Protocol for a Cross-Sectional Study”, the first sentence of the Methods “In this cross-sectional study, students enrolled in a School of Nursing located at a University in Ontario, Canada…”, and as outlined in Figure 1, this is a protocol for a cross-sectional study and not a prospective study. Therefore, we are not following students across the four years of their degree; rather, we are cross-sectionally assessing 80 students in each year of nursing studies (total students=320) at one point in time. A prospective study of this nature would be interesting, however, we agree with the Reviewer that the resources required for such a study would be heavy. It is important to note, that a cross-sectional study of nursing students meets our stated objective of: “The goal of this research is to examine multiple lifestyle factors (including PA, nutrition, sleep, and stress) and determine whether these factors are associated with metabolic health in full-time undergraduate nursing students.”.

Therefore, the logistics of the study will include 320 student visits (Figure 1: study visit 1), and then the return of equipment to the laboratory 7 days later upon completion of accelerometry and dietary log collection. The return of the equipment is a simple drop off- and will not require a formal visit. We have recruited a graduate student dedicated to the study completion, multiple undergraduate research assistants, in addition to the Investigators of the study to assist with data collection. Therefore we are confident that we have the resources to deliver the 320 student visits that include testing.

**5. What is the time-frame of the funded project?**

RESPONSE: Ideally, our timeframe will be one academic year; however, if we observe a delay in study recruitment and/or study testing, we will expand our timeframe to two academic years. Our funding source is flexible with the funding timeline (especially given the COVID-19 pandemic). We have added the following sentence into the Methods (page 9) to clarify our time-frame: “Ideally, we will recruit all participants during one academic year. If required, we will extend our recruitment and data collection over a second academic year.”
